# Temperament Upregulates Mitochondrial Enzymes and Negatively Affects Myofibrillar Fragmentation in Beef of Excitable *Bos taurus indicus* Cattle

**DOI:** 10.3390/metabo15010024

**Published:** 2025-01-07

**Authors:** Patricia Maloso Ramos, Eduardo Francisquine Delgado, Ana Cláudia da Silva, Nara Regina Brandão Cônsolo, Vinicius Laerte Silva Herreira, João Marcos Bovetto de Campos Valim, Fernanda Maria Marins Ocampos, Luiz Alberto Colnago, Saulo da Luz e Silva

**Affiliations:** 1 Departamento de Zootecnia, Faculdade de Zootecnia e Engenharia de Alimentos (FZEA/USP), Universidade de São Paulo, Pirassununga 13.635-900, SP, Brazil; sauloluz@usp.br; 2 Departamento de Zootecnia, Escola Superior de Agricultura “Luiz de Queiroz” (ESALQ/USP), Universidade de São Paulo, Piracicaba 13.418-900, SP, Brazil; efdelgad@usp.br (E.F.D.);; 3 Departamento de Nutrição e Produção Animal, Faculdade de Medicina Veterinária e Zootecnia (FMVZ/USP), Universidade de São Paulo, Pirassununga 13.635-900, SP, Brazil; nara.consolo@usp.br (N.R.B.C.); vinicius.laerte@usp.br (V.L.S.H.); jmvalim15@gmail.com (J.M.B.d.C.V.); fmmocampos@gmail.com (F.M.M.O.); 4 Empresa Brasileira de Pesquisa Agropecuária (EMBRAPA Sudeste), São Carlos 13.561.206, SP, Brazil; luiz.colnago@embrapa.br

**Keywords:** calpastatin, energy metabolism, glycolytic, mitochondrial, oxidative, stress

## Abstract

Background: *Bos taurus indicus* cattle is known to be temperamental and to produce beef with greater variability in terms of quality compared to beef of *Bos taurus taurus*. Cattle adaptability and resilience are of great importance to sustain beef production worldwide. Objective: The study aimed to understand early post-mortem metabolites among muscles with different fiber types profile of calm and excitable Nellore, as well as its relationship with fragmentation of beef aged up to 28 d. Methods: Animals were evaluated based on chute score and exit velocity to calculate a temperament index, which was used to classify them as calm or excitable. At slaughter, the pH and temperature declines of *Triceps brachii* (TB) and *Longissimus lumborum* (LL) were measured, muscles were sampled, and aged up to 28 d. Metabolites were determined, and sarcomere length and myofibrillar fragmentation index (MFI) were quantified. Metabolomics data were analyzed using a multivariate approach, while other traits were investigated through ANOVA. Results: The pH decline was affected by all three fixed effects investigated (temperament × muscle × time post-mortem: *p* = 0.016), while temperature decline was affected by muscle × time (*p* < 0.001). Metabolites differed among muscles and cattle temperament, with excitable cattle showing greater taurine abundance in LL, as well as greater creatine in TB 1 h post-mortem, based on the volcano plot. Sarcomere length and MFI results revealed faster and limited tenderization in excitable cattle beef. Conclusions: Altogether, results emphasized the upregulation of mitochondrial enzymes and reduced tenderization as determinants of inferior beef quality after prolonged aging in excitable cattle.

## 1. Introduction

*Bos taurus indicus* breeds and its crosses contribute to a substantial proportion of meat produced worldwide and have the potential to meet the growing global demand for high-quality protein for human consumption. The Brahman and Nellore breeds are two of the most prominent representatives of the *Bos taurus indicus* breeds, playing a crucial role in the livestock industries of Brazil, Australia, the United States and Central America. Their importance stems largely from their remarkable adaptation to tropical and subtropical environments, including traits such as heat tolerance, parasite resistance, and the efficient utilization of low-quality forage [1]. However, a couple of challenges do exist related to raising this biological type. The first one, dearly investigated in the 80s to 90s, was the temperamental characteristic of this cattle [2,3,4]. The second one learned long ago from various studies involving *Bos taurus indicus* beef tenderization was related to the importance of calpastatin as the inhibitor of proteolysis, therefore reducing extension and slowing beef tenderization [5,6,7]. From those investigations, aligned with increasing interest in producing beef with superior quality, most quality assurance programs implemented worldwide disregarded this biological type. Beef from *Bos taurus indicus* and its influenced cattle was associated with inferior quality.

Researchers nowadays are trying to elucidate the mechanisms that correlate this ability to thrive under climate changes and the physiological adaptations, mostly related to energy balance in vivo that translate to early post-mortem, which will drive the tenderization process [8], in the hope of enhancing beef quality. Mitochondrial function early post-mortem in *Longissimus* of Brahman definitively contributes to ATP production, delayed pH decline, and calpain activation when compared to Angus [9]. Interesting is the fact that it is not a coincidence that the scenario worsens when raising excitable *Bos taurus indicus* cattle. As was also reported before, the activation of the stress cascade can increase calpastatin activity by post-translational modification and altering expression [10,11].

Early post-mortem metabolism differs among skeletal muscles based on their properties, which include mitochondrial abundance, sarcoplasmic reticulum sizes and distribution as well as calpain-calpastatin contents [12]. For example, muscles with a greater contribution of oxidative fibers have a greater content of mitochondria and calpain-calpastatin to match the demands of energy to sustain contraction as well as enhanced protein turnover [12]. Therefore, it is expected that *Triceps brachii* and *Longissimus*, classified as intermediate and white muscles, respectively [13], would rely on different pathways to sustain energy demands in vivo and post-mortem. Additionally, the response of stress altering the metabolism of those skeletal muscles of *Bos taurus indicus* cattle is unknown.

Therefore, our objective was to understand the early post-mortem metabolites differences among muscles with distinct fiber type profiles of calm and excitable Nellore animals, as well as its relationship with the myofibrillar fragmentation index (MFI) of beef aged up to 28 d. The hypothesis driving the study is that the stress response will negatively influence beef tenderization by upregulating mitochondria in excitable cattle.

## 2. Materials and Methods

### 2.1. Animals

All procedures involving the animals in this study were previously approved by the local Committee of Ethics in Animal Use (protocol number 6493190121).

The animals used in this study were part of an experiment designed to evaluate growth traits of immunocastrated (IM; n = 30) and non-castrated (NC; n = 30) Nellore males during the finishing phase. Upon arrival at the feedlot (average body weight of 350 ± 30 kg and 20 months old), all animals (n = 60) were evaluated for temperament (as described below). All animals (n = 60) were housed in a feedlot facility and fed for approximately 100 days. The IM group received two doses of the immunocastration vaccine (Anti-GnRH, Bovipriva^®^, Pfizer Animal Health, São Paulo, Brazil) 62 d before the beginning of the finishing period, and the second dose when it arrived at the feedlot, according to the manufacturer’s instructions.

### 2.2. Temperament Evaluation and Index Calculation

When animals arrived at the feedlot, the temperament evaluation was conducted. The temperament was evaluated by the combination of two tests: (1) the chute score (CS) and (2) the exit velocity (EV). The first test was a visual and subjective evaluation in which a trained professional gave a score on a scale from 1 to 4 as soon as the animal entered the chute (1 = no movement, with head, ears and tail relaxed; 2 = some movement, with the head and the ears upright; 3 = frequent but not vigorous movement and possible vocalization; and 4 = constant and strong movement, frequent vocalization, and showing great resistance; adapted from [4]). The second test was performed objectively by recording the time elapsed between the animal exiting the chute and the animal reaching a 5.4 m marked distance. The time was recorded by an observer, with the release of the chronometer as soon as the animal’s nose was observed leaving the chute and stopping the chronometer as soon as the rear touched the marked distance. The EV was reported in meters per second (m/s; adapted from [14]). The temperament index (TI) was calculated by numerically averaging both tests, as shown in the following formula: TI = (CS + EV)/2, as previously reported [15].

Based on the TI information, the overall average and standard deviation were calculated for all animals and a group of twenty-two (n = 22) animals (10 NC and 12 IM) was selected to represent two groups with distinct temperaments: calm (n = 11; 5 NC and 6 IM) and excitable (n = 11; 5 NC and 6 IM).

### 2.3. Slaughter and Sampling

Animals were slaughtered as soon as they reached a minimum of 5 mm of backfat thickness measured by ultrasound in the *Longissimus lumborum* (LL) muscle between the 12th and 13th ribs. The slaughters were conducted in an experimental slaughterhouse belonging to the University of Sao Paulo (*campus* Pirassununga, SP, Brazil) that works under the State Inspection Services (SISP, Brazil). Due to the technical limitations of the facility, there was a limited number of animals that could be slaughtered per day, following a previous classification based on the backfat thickness. Therefore, multiple slaughter dates were necessary to slaughter all animals, and this information was added to the statistical analysis to account for that variation properly.

Since the feedlot was located near the slaughter facility (~400 m), the animals were fasted in the feedlot and transported by truck on the morning of slaughter day to the slaughterhouse. Fasting was at least 12 h prior to transport, with free access to water.

#### 2.3.1. Muscle pH and Temperature Declines

Two muscles were previously selected to be sampled from each pre-selected animal: the LL and the *Triceps brachii* (TB) muscles. Those muscles were chosen based on their fiber type compositions [13] and anatomic locations. While the LL is a more glycolytic muscle located in the caudal portion of the beef carcass, the TB is a more oxidative (classified as intermediate) muscle located in the cranial and ventral parts. Both muscles were assessed using pH and temperature decline measures.

For pH measures, the pH meter was previously calibrated and maintained in the same room as the carcasses. Therefore, the buffers were kept in the same room as the carcasses, as well. Calibration was performed using buffers 4 and 7, and buffer 7 also served as an indicator of accuracy, with a deviation of ± 0.05 considered the threshold for repeated calibration. The pH decline was measured directly by the insertion of a portable pH meter in the LL and TB muscles (Hanna, model HI99163, Hanna Instruments, Barueri, SP, Brazil) during the first 24 h post-mortem (1, 3, 6, 9, and 24 h). The temperature measurement was performed simultaneously by the insertion of the thermometer near the pH meter. The pH meter probe was always inserted in a new position, avoiding damaged tissue and always in the same half-carcass of each animal.

#### 2.3.2. Muscles Samples, Sarcomere Length, and Myofibrillar Fragmentation Index Determination

Small samples were collected at 1 h post-mortem from the LL and TB and immediately frozen in liquid nitrogen. At 24 h post-mortem, the LL and TB were excised from the carcasses and fabricated into a steak, sampled in 5 distinct anatomical positions to represent the entire surface and allow the sarcomere length determinations. Small pieces of the remaining steak were cut and separately vacuum packaged and aged in a dark, cold room (4 °C) for 1, 7, 14, and 28 d. After each aging time, the pieces were further cut into small cubes, frozen with liquid nitrogen and transferred to an ultra-freezer (−80 °C) for further myofibrillar fragmentation index analysis (MFI) [16].

#### 2.3.3. Metabolomic Analysis

Samples collected from both muscles at 1 and 24 h post-mortem that were stored in the ultra-freezer were further destined for metabolomics analysis. Metabolites were extracted as previously described [17] and analyzed through nuclear magnetic resonance spectrometry (^1^H-NMR). ^1^H-NMR analyses were performed at 300 K on a Bruker AVANCE III HD 600 NMR spectrometer (Bruker, Karlsruhe, Germany), operating at 14.1 T, observing ^1^H and ^13^C at 600.13 and 150.90 MHz, respectively, and equipped with a 5 mm multinuclear direct detection probe with z-gradient. A QC sample, composed of a pool of all samples, was used to calibrate the 90° pulse length, adjust the gain to all samples, determine the offset of the water signal for the water suppression, and periodically monitor acquisition. Spectra were acquired using a 1D NOESY-presat (*noesypr1d*) pulse sequence on a spectral width of ~20 ppm. Each ^1^H-NMR spectrum consisted of 128 scans, with the following parameters: 0.37 Hz/point; acquisition time (AQ) 2.73 s; relaxation delay (RD) 4.0 s; 90° pulse width (PW) 13.08 μs with receiver gain of 128. Spectra were processed using the TopSpin3.5 software v 4.4.1 (Bruker). All ^1^H-NMR chemical shifts were observed in ppm related to the DSS signal at 0.00 ppm as an internal reference, and an exponential line broadening of 0.3 Hz was applied. After the Fourier transformation, spectra were automatically phased, and baselines were automatically corrected with manual correction when necessary. Metabolites were assigned based on the chemical shifts, signal multiplicities, and integrals, always in comparison to the literature. After processing the NMR data, metabolites were identified and quantified using the Chenomx software v 10.0 (Chenomx Inc., Edmonton, AB, Canada).

### 2.4. Statistical Analysis

Muscle metabolomic data were analyzed using MetaboAnalyst 6.0 (http://www.metaboanalyst.ca/) through Volcano Plot and enrichment analysis. The results are presented by muscle and at each time post-mortem separately. For the pH and temperature declines, as well as MFI, data was analyzed using an analysis of variance in a randomized block design, in which the model included fixed effects of animal temperament (calm vs. excitable), muscle (LL vs. TB), time post-mortem, as well as the doubles and triple interactions. The sexual category was not the primary focus of this study; however, its effect on the evaluated traits was tested. As no significant statistical effect was observed for this factor, the sexual condition was considered a random effect along with slaughter dates and animals. Time was considered a repeated measure, and the co-variance structure considered the subject as being the animal within muscle and time interaction. For sarcomere length, the time effect was removed from the model. The statistical software used was SAS On Demand for Academics v 3.8 (Cary, NC, USA). The significance observed is reported and discussed accordingly.

## 3. Results

### 3.1. Muscles pH and Temperature Declines

**Figure 1:**
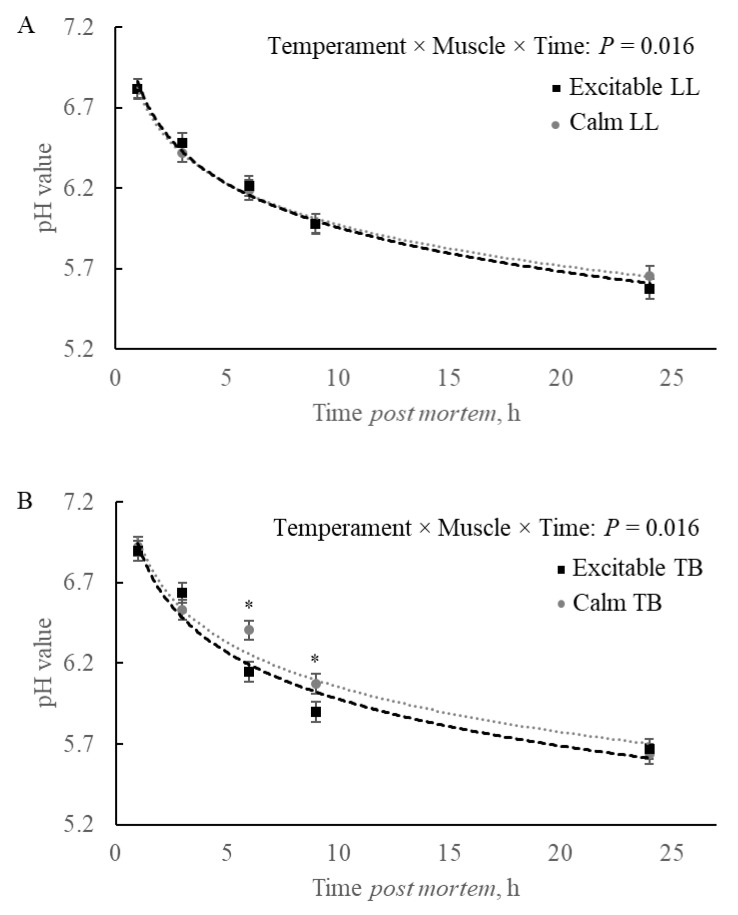
pH declines post-mortem in (**A**) *Longissimus lumborum* (LL) and (**B**) *Triceps brachii* (TB) muscles of Nellore carcasses. * *p* ≤ 0.05.

For the pH decline, it was observed a triple interaction between temperament × muscle × time post-mortem (*p* = 0.016; Figure 1A,B). Comparing groups formed by the temperament and muscles separately and during the time, a post-mortem showed that no difference was observed within the LL muscle (Figure 1A). However, when the comparison was among groups within the TB muscle, the excitable group showed a faster pH decline, with differences observed at 6 and 9 h post-mortem, without differences at initial (1 and 3 h post-mortem) or final pH value (24 h; Figure 1B).

**Figure 2:**
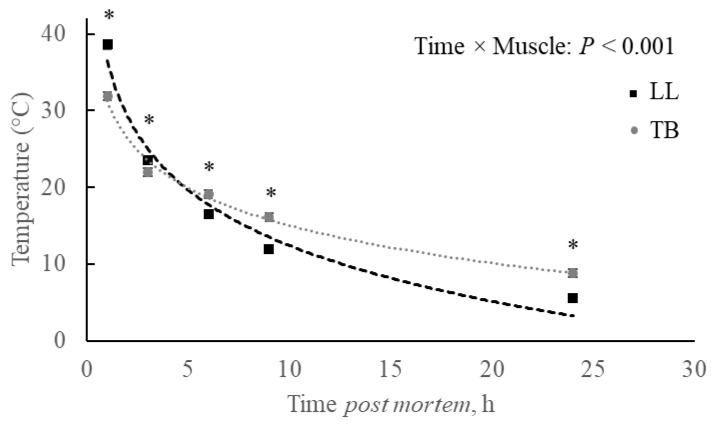
Temperature declines post-mortem in *Longissimus lumborum* (LL) and *Triceps brachii* (TB) muscles of Nellore carcasses. * *p* ≤ 0.05.

The temperature decline was not influenced by the triple interaction, but it was influenced by the double interaction between temperament × muscle (*p* = 0.064) and muscle × time (*p* < 0.001; Figure 2). Considering the overall average (all times post-mortem taken together) for calm LL, the temperature was lower (18.75 ± 0.304 °C) than the temperature observed for calm TB (19.63 ± 0.304 °C; *p* < 0.05), with the excitable LL and TB showing intermediate values and without difference from calm animals. On the other hand, when temperature decline was compared between LL and TB during the post-mortem period, the LL muscle started with greater temperature, which stayed higher at 3 h post-mortem. After this point, an inversion in the pattern was observed, and the LL lost temperature faster than the TB, which showed greater values at 6, 9, and 24 h post-mortem (Figure 2).

### 3.2. Muscles Metabolism

**Figure 3:**
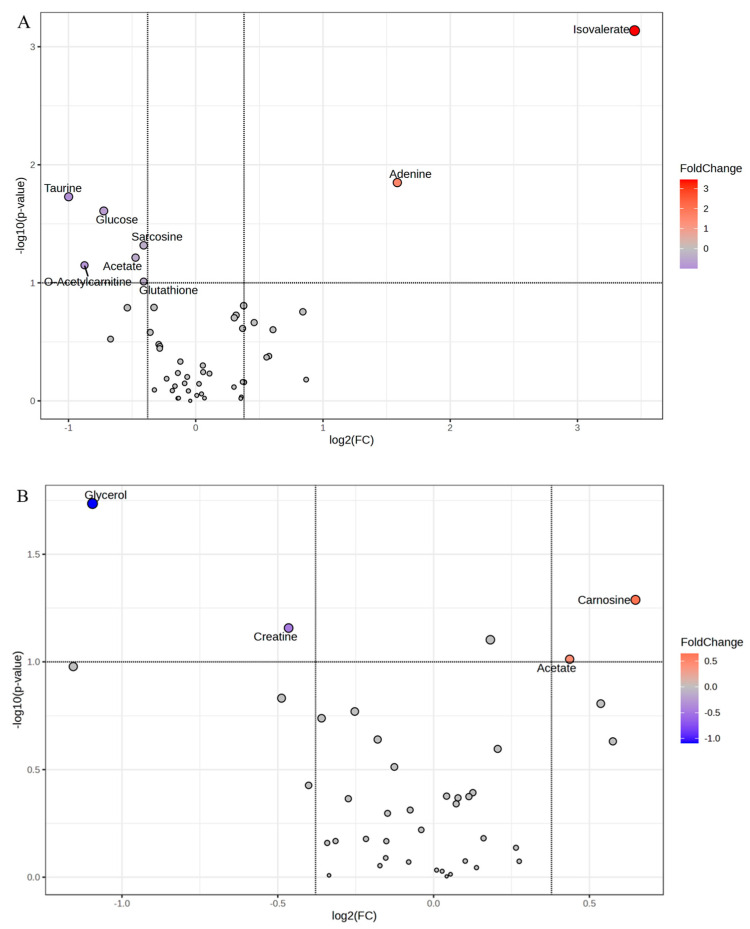
Volcano plot of metabolites in (**A**) *Longissimus lumborum* (LL) and (**B**) *Triceps brachii* (TB) muscles at 1 h post-mortem of Nellore cattle classified as excitable (blue) or calm (red).

The 1 h post-mortem LL volcano plot revealed that metabolites isovalerate and adenine were greater in calm animals, while taurine, glucose, sarcosine, acetate, o-acetyl carnitine and glutathione were greater in excitable animals (Figure 3A). On the other hand, the volcano plot representing the TB metabolites at 1 h post-mortem showed greater carnosine and acetate in calm animals, while glycerol and creatine were more abundant in the muscles of excitable animals (Figure 3B).

**Figure 4:**
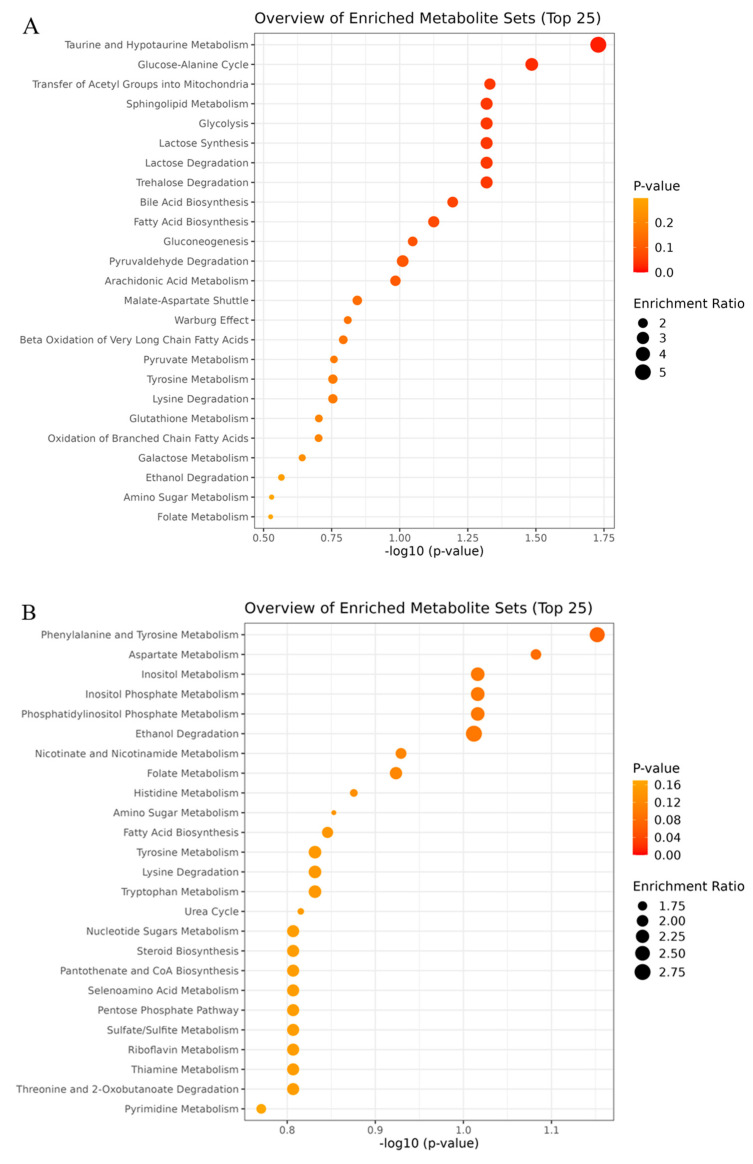
Overview of enriched pathways in (**A**) *Longissimus lumborum* (LL) and (**B**) *Triceps brachii* (TB) muscles at 1 h post-mortem of Nellore cattle classified as excitable or calm.

The overview of enriched pathways for the LL 1 h post-mortem showed that taurine and hypotaurine metabolism was the most significant (*p* = 0.019) pathway to differentiate groups of temperament (Figure 4A). The metabolite that contributed to the differentiation was taurine, which was relatively more abundant (*p* = 0.019) in the excitable muscle compared to that of calm animals. The second significant pathway, the glucose-alanine cycle (*p* = 0.033), was mostly driven by the relative abundance of D-glucose and glutamate. While D-glucose showed greater abundance (*p* = 0.025) in the LL of excitable animals, no differences were associated with the abundance of glutamate among temperament groups. The third pathway that contributed to the differentiation of groups was the transfer of acetyl groups into mitochondria (*p* = 0.047). In this group, the metabolites involved were D-glucose, malate and ATP. However, only the D-glucose showed greater (*p* = 0.025) relative abundance in the LL of excitable animals.

For TB at 1 h post-mortem, the most significant pathway involved in the differentiation of the animal’s temperament was the phenylalanine and tyrosine metabolism (Figure 4B). The metabolites that most contribute to this pathway were phenylalanine, which was greatly concentrated (*p* = 0.079) in the TB of calm animals, and glutamate (*p* = 0.147). The second pathway, aspartate metabolism (*p* = 0.082), involved five metabolites. The acetate relative abundance was greater (*p* = 0.097) in TB of calm animals, while glutamate, L-arginine, glutamine, and malonate were all involved with similar relative abundance among groups of temperament.

**Figure 5:**
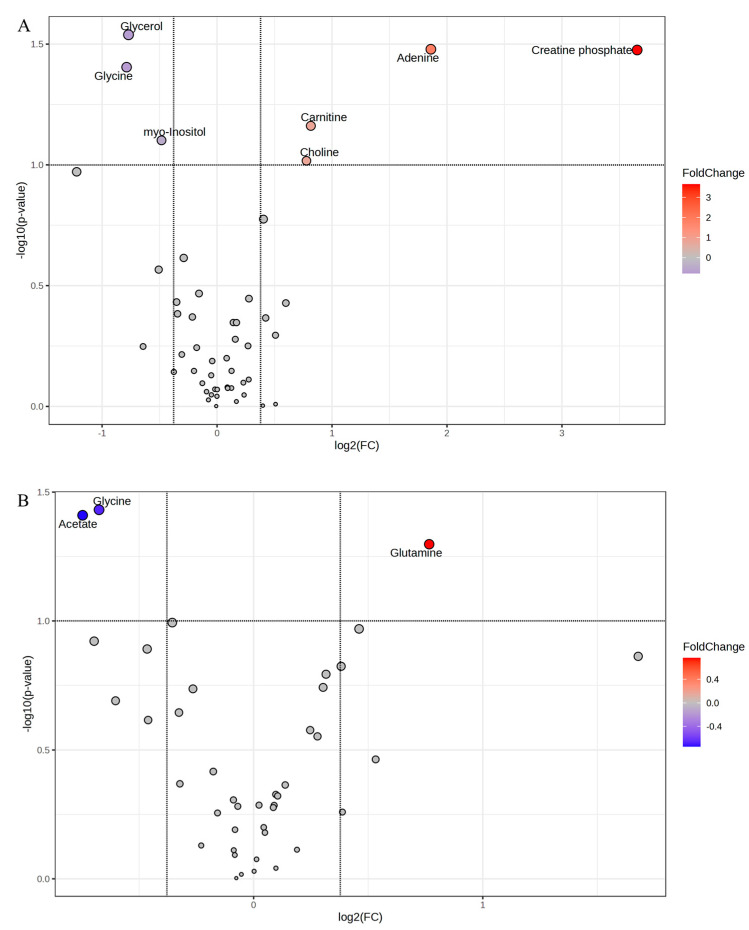
Volcano plot of metabolites in (**A**) *Longissimus lumborum* (LL) and (**B**) *Triceps brachii* (TB) muscles at 24 h post-mortem of Nellore cattle classified as excitable (blue) or calm (red).

As expected, the scenario changed completely when the metabolites were analyzed in muscle samples collected after 24 h post-mortem. The Volcano plot representing LL at 24 h (Figure 5A) showed that glycerol, glycine, and myo-inositol were greater in excitable animals, while creatine phosphate, adenine, carnitine, and choline were greater in the LL muscle of calm animals. Interestingly, glycerol was also greater in the TB muscle of excitable animals at 1 h post-mortem. For the TB 24 h post-mortem, the volcano plot revealed that glycine (*p* = 0.037) and acetate (*p* = 0.038) were greater in muscle of excitable animals, while glutamine was greater (*p* = 0.050) in muscle of calm animals (Figure 5B). Another interesting observation was that acetate was greater in TB 1 h post-mortem from calm animals.

**Figure 6:**
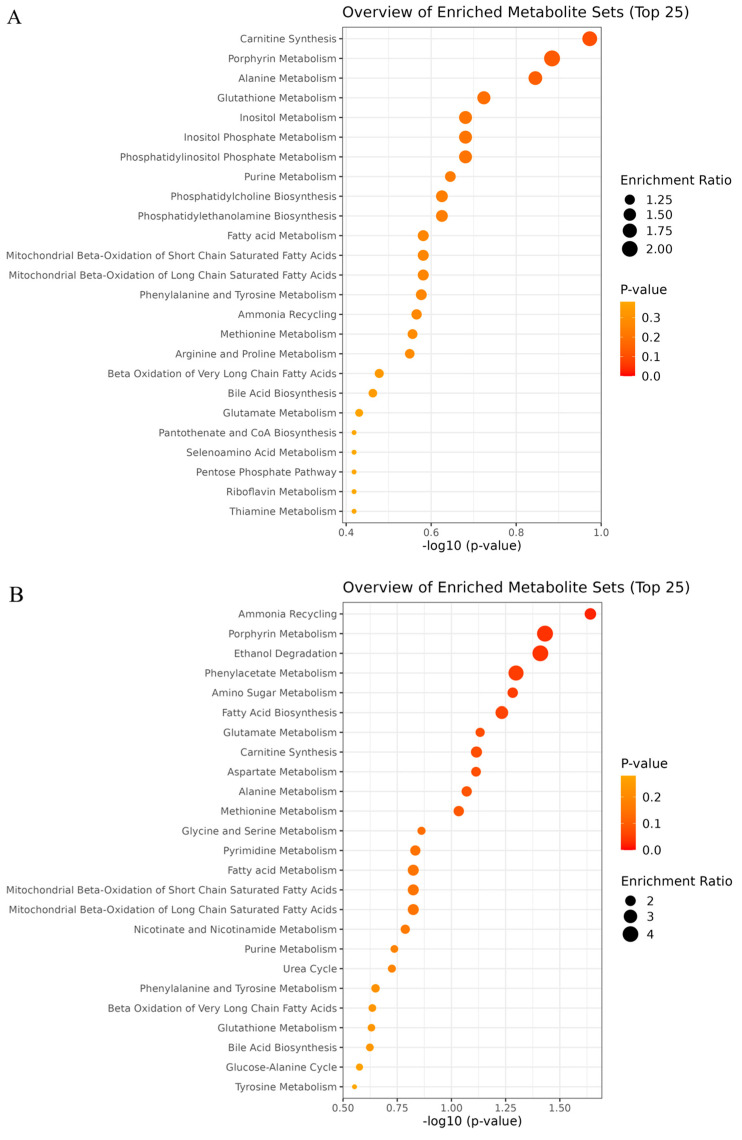
Overview of enriched pathways in (**A**) *Longissimus lumborum* (LL) and (**B**) *Triceps brachii* (TB) muscles at 24 h post-mortem of Nellore cattle classified as excitable or calm.

The overview of enriched pathways using metabolites of LL 24 h post-mortem revealed that carnitine synthesis was the most significant (*p* = 0.106) pathway to differentiate groups of temperament (Figure 6A). Within this pathway, the metabolites involved were glycine, which showed greater abundance (*p* = 0.040) in LL of excitable animals; L-carnitine, which showed greater abundance (*p* = 0.069) in LL of calm animals; succinate and NADH, without differences among groups. The second pathway involved in the segregation of the groups was the porphyrin metabolism (*p* = 0.130). In LL 24 h post-mortem, the metabolites involved in this pathway were glycine and NADH.

The metabolites identified in the TB 24 h post-mortem, when analyzed based on the enriched pathways, showed that the ammonia recycling pathway was the most significant (*p* = 0.023) in differentiating the groups of temperament (Figure 6B). Within this pathway, the metabolites involved were glycine, glutamine, pyruvate, glutamate and ADP. While glycine showed greater (*p* = 0.037) relative abundance in TB of excitable animals, glutamine was greater (*p* = 0.050) in TB of calm animals. No differences were observed among groups in terms of the relative abundance of pyruvate, glutamate, and ADP. Similar to the observed in the LL, the second pathway most significant in the differentiation of the groups was porphyrin metabolism (*p* = 0.037). In this pathway, the metabolite involved was glycine, greater (*p* = 0.037) in TB of excitable cattle.

### 3.3. Sarcomere Length

**Figure 7:**
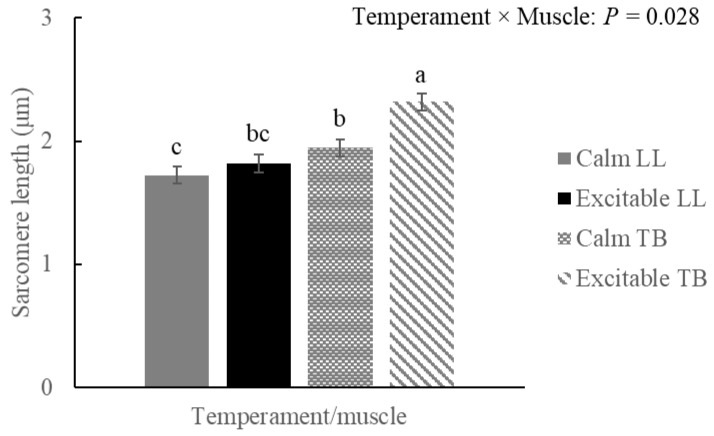
Sarcomere length (μm) in *Longissimus lumborum* (LL) and *Triceps brachii* (TB) muscles at 24 h post-mortem of Nellore cattle classified as excitable or calm. ^a–c^ *p* ≤ 0.05, different letters representing statistical differences.

An interaction effect between temperament × muscle (*p* = 0.028) and a longer sarcomere length was observed in the TB of excitable animals (Figure 7). The comparison within muscle showed a difference among temperaments for TB (*p* = 0.001) and no difference among groups within LL. However, shorter sarcomere length (*p* = 0.018) was observed in LL of calm animals when compared to the sarcomere in TB of the same temperament group.

### 3.4. Myofibrillar Fragmentation Index (MFI)

**Figure 8:**
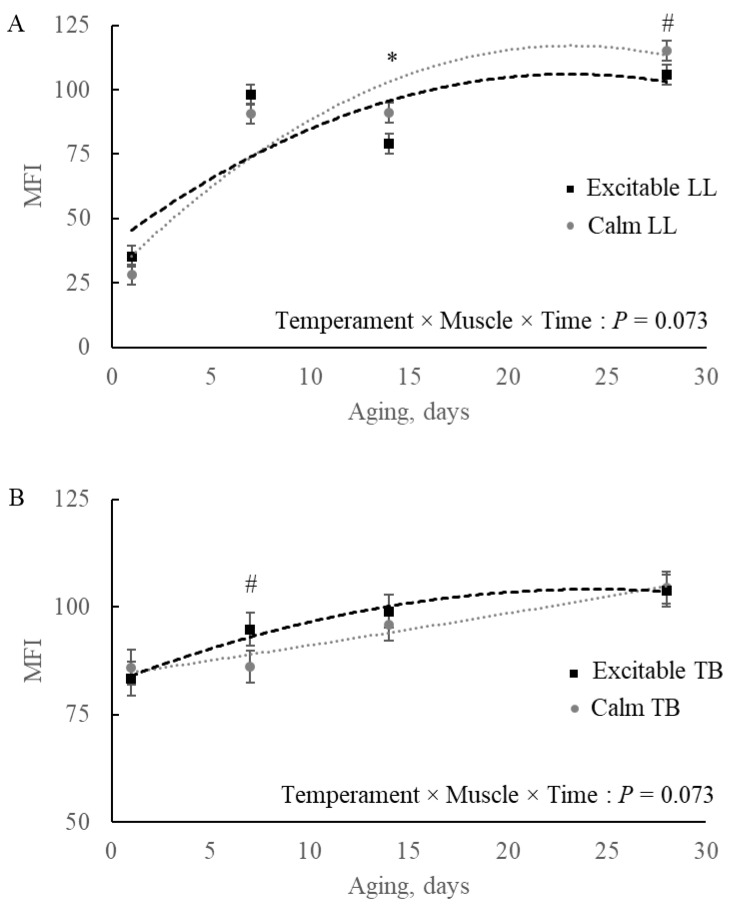
Myofibrillar fragmentation index (MFI) in (**A**) *Longissimus lumborum* (LL) and (**B**) *Triceps brachii* (TB) muscles during 28 d aging in beef of Nellore cattle classified as excitable or calm. * *p* ≤ 0.05; # 0.05 < *p* ≤ 0.10.

It was observed a triple interaction effect temperament × muscle × time (*p* = 0.073; Figure 8) for MFI. For LL, the differences were observed at 14 and 28 d of aging, with beef of calm animals showing greater fragmentation than that of excitable animals (Figure 8A). For TB, on the other hand, the greater fragmentation was observed at 7 d of aging and was related to the beef of excitable cattle, although the difference disappeared at later aging periods (Figure 8B).

**Figure 9:**
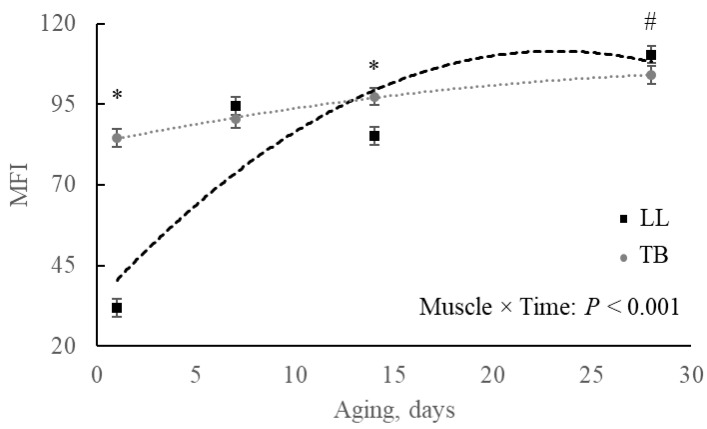
Myofibrillar fragmentation index (MFI) in *Longissimus lumborum* (LL) and *Triceps brachii* (TB) muscles during 28 d aging in beef of Nellore cattle. * *p* ≤ 0.05; # 0.05 < *p* ≤ 0.10.

The comparison of MFI progression among muscles during the aging period revealed that the LL showed less fragmented myofibrillar proteins at initial aging (*p* < 0.001), reaching the same fragmentation as TB by day 7 and surpassing the fragmentation observed in TB by the final aging day (*p* = 0.062; Figure 9).

## 4. Discussion

As a multifactorial process, muscle-to-meat conversion undergoes a series of biochemical and physical changes during the first 24 h after the animal is harvested. Muscles with distinct fiber type profiles, such as LL and TB, respond differently to stress before slaughter due to their ability to balance energy production and utilization, affecting pH decline. Those muscles have inherent differences in terms of organelle sizes and distribution across muscle fibers, such as greater development of sarcoplasmic reticulum in more glycolytic LL and greater mitochondrial abundance in TB [18]. In this context, the muscles of excitable animals can further adapt and modulate in vivo metabolism that will translate to post-mortem, which can be verified in the rate of pH decline. The pH decline observed in the present study (temperament × muscle × time: *p* = 0.016) explored within muscles revealed no differences among animals’ temperaments within LL (Figure 1A). However, TB was impacted by animal temperament (Figure 1B), with a faster rate associated with excitable animals. The faster pH decline in TB of excitable cattle could be attributed to faster ATP production as well as faster hydrolysis during the establishment of *rigor mortis* around 6 and 9 h post-mortem compared to TB of calm animals. Called before a ‘mitochondrial treason,’ the greater use of ATP under oxygen deprivation by more oxidative muscle [19] could be exacerbated in the muscle of excitable cattle as a response to the slaughter stress, increasing the activity of several enzymes in the sarcoplasm as well as mitochondrial.

The change in the muscle metabolism that resulted in faster pH decline caused by the differential stress cascade in the excitable cattle is further corroborated by the metabolites. At 1 h post-mortem, in the TB of excitable animals, greater glycerol and creatine abundance were observed compared to the TB of calm animals, which presented more carnosine and acetate (Figure 3B). The activation of the stress cascade, for example, starting from the catecholamines binding the beta-adrenergic receptor in the sarcolemma, which would stimulate the G protein dissociation, promote adenylyl cyclase activation, increasing the cyclic AMP (cAMP) concentration in sarcoplasm, with consequences in the energy balance. In the case of TB early post-mortem, the cAMP functions as an intracellular messenger, signaling greater energy demand to sustain the greater muscle contraction after stimuli, and to accomplish that, the muscle would rely on the creatine kinase system. The more oxidative metabolism of the TB allowed the creatine accumulation, as was observed in high-pH LL of Nellore cattle [20], which points to dephosphorylation of phosphocreatine by sarcoplasmic creatine kinase to supply ATP under high-energy demand. The production of sarcoplasmic phosphocreatine under high-energy demand depends on the mitochondrial creatine kinase, therefore coupled to the mitochondrial metabolism.

The overview of enriched pathways for TB muscle and 1 h post-mortem showed most pathways correlated to the amino acids and energy metabolism as important to differentiate groups of temperament (Figure 4B). The first most significant pathway was the phenylalanine and tyrosine metabolism, in which phenylalanine was greater (*p* = 0.079) in the muscles of calm animals. The activation of this metabolism is evidence of protein breakdown after a prolonged period of muscle contraction, as is the case during early post-mortem. While the activation of this pathway can be associated with fiber types I and II, it was previously demonstrated that in human skeletal muscles with lower glycogen levels, those metabolites accumulate faster [21]. The second pathway that differentiated both temperament groups was the aspartate metabolism, with greater (*p* = 0.097) acetate in TB of calm animals.

It is well known that the temperature contributes to modulating the rate of pH decline and, therefore, it must be considered. In the present study, no difference was observed regarding temperature decline based on animal temperament. However, the rate of temperature decline could have affected the rate of pH decline in both muscles since it was observed a similar pattern for this trait among temperaments within the muscles (muscle × time: *p* < 0.001; Figure 2). Therefore, the temperature decline patterns according to muscles were probably a reflection of the muscle position in the carcass, as well as the fat insulation. Since TB is in the forequarters of the carcass, it makes sense that it started (1 h) at a lower temperature (*p* < 0.05), which was expected due to the lower muscle mass available to keep the physiological temperature. However, as time passed and the carcasses were put in the cold room that had the cold air inlet from above, six hours post-mortem was enough to result in the cooler (*p* < 0.05) LL, which remained cooler through the end (24 h) of carcasses cooling period. Important to notice that this difference most probably contributed to the altered TB muscle pH decline at *rigor mortis* onset between temperaments.

Although no differences were observed among temperaments within LL pH decline, there were interesting differences related to muscle metabolites profile evaluated at 1 h post-mortem. The most significant finding for LL was related to taurine abundance (Figure 3A) and the relevance of taurine and hypotaurine metabolism to differentiate those groups of temperament (Figure 4A). In this context, the LL of excitable cattle showed greater abundance (*p* = 0.018) of taurine compared to that of calm animals. This remarkable finding amplifies the current knowledge related to muscle energy use and its regulation in the context of exacerbated stress response. Taurine is an amino acid related to multiple protective functions within the skeletal muscle, including but not limited to the protection of oxidative stress [22]. From a meat science perspective, supplementation with taurine in a heat-stressed broilers’ diet improved mitochondrial function, enhancing protection from oxidative attack, as well as increasing citrate synthase activity within glycolytic breast muscle, enhancing poultry meat quality [23]. Similarly, pigs fed taurine showed enhanced mitochondrial biogenesis and function, with a consequent decrease in *Longissimus* muscle glycolytic potential, resulting in greater pork quality [24]. The authors discussed the reduction of the glycolytic potential as being a consequence of a fiber-type conversion from the more glycolytic profile towards a more oxidative contribution since taurine stimulated the calcineurin/NFATc1 pathway. Therefore, increased taurine concentration within a glycolytic muscle was beneficial for meat products from monogastric animals. In the case of excitable cattle, the novel finding of taurine related to early post-mortem muscle-to-meat conversion points to a natural activation of a pathway to preserve muscular function after chronic stress stimuli. In this case, it is hypothesized that after the persistency of the sympathoadrenal system and hypothalamic-pituitary-adrenal axis activation in those (excitable) animals, the skeletal muscle adapted the metabolism to rely on the benefits of the taurine pathway to both energy signaling, as well as to oxidative damage protection.

While the contribution of taurine in the protection of the muscle cell against oxidative stress could be corroborated by the glutathione, which was also more abundant in the LL of excitable cattle (Figure 3A), the contribution in the energy signaling pathway is more intricate. It was previously shown that taurine is involved in the activation of the main energy sensor in skeletal muscle, the AMP-dependent kinase (AMPK) [25]. In this case, authors demonstrated that taurine could stimulate phospholipase C to increase calcium influx by its interaction with the taurine transporter, activating the AMPK by phosphorylation, which would stimulate the expression of mitochondrial proteins and myoglobin, among others. To the best of our knowledge, it is the first time that the taurine pathway has been described in the context of skeletal muscle energy regulation in beef cattle. The activation of this pathway could help to explain the greater occurrence of atypical dark and dark beef related to excitable *Bos taurus indicus* cattle, a biological type that is more resilient to environmental stresses but is also considered more temperamental. Nevertheless, taurine was previously identified as being upregulated (*p* = 0.042) in dark cutting compared to normal beef [26].

The relevance of the stress to model LL metabolites was further evidenced by the second most significant pathway, the glucose-alanine cycle (*p* = 0.033), to differentiate temperament groups. The greater abundance of D-glucose (*p* = 0.025) in LL of excitable cattle points to the greater mobilization of glycogen, as well as the up-regulation enzymes involved in glycogenolysis and glycolysis. Along with that, the transfer of acetyl groups into mitochondria was the third most significant pathway (*p* = 0.047) that contributed to differentiating the groups. A faster rate of glycolysis would result in greater accumulation of glucose and acetyl-CoA, enhancing ATP availability in the cytosol. Acetyl-CoA can be generated in the cytosol by acetate and then transferred back to mitochondria, which is crucial for maintaining TCA cycle activity [27].

Interestingly, LL of excitable animals after 24 h post-mortem showed greater glycerol, glycine, and myo-inositol accumulation (Figure 5A). Since glycerol and myo-inositol were more abundant in TB of excitable cattle at 1 h post-mortem as well, it looks like that, for these cattle, we could order the rate of post-mortem oxidative metabolism, being faster in TB than in LL. Independent of the rate of post-mortem metabolism in excitable cattle, in muscles of calm animals, the metabolite profile was different and revealed the greater abundance and persistency of adenine in LL at 24 h post-mortem, along with creatine phosphate, which together indicate the lower pressure into the phosphagen system to sustain ATP concentration earlier, which resulted from anaerobic glycolysis. According to traditional knowledge related to muscle-to-meat conversion, the accumulation of adenine in post-mortem muscle is expected. In dark cutting compared to normal pH, beef adenine was downregulated in the former [26].

Porphyrin metabolism was the second enriched pathway to significantly differentiate temperament groups for both muscles at 24 h post-mortem (Figure 6A,B), with glycine as the differential metabolite involved in this pathway and more abundant in the muscle of excitable cattle. Porphyrin is an intermediary compound formed by the combination of succinyl-CoA and glycine, which will receive an iron atom in its center to become the heme group [28]. A greater heme group could be associated with previously reported taurine abundance once the muscles of excitable cattle modified the metabolism towards a more oxidative capacity, potentially increasing myoglobin content in LL (i.e., as observed in dark-cutting beef). Interestingly, porphyrin, as well as heme, depends on an energy-dependent transporter to cross the membrane, and its accumulation within the cell causes toxicity [29]. Furthermore, porphyrin is involved with calcium release from the sarcoplasmic reticulum to the sarcoplasm by interacting with the ryanodine receptor in an ATP-dependent manner [30]. In this case, greater porphyrin metabolism associated with excitable cattle could promote earlier calcium release to sarcoplasm and, therefore, enhance initial myofibrillar fragmentation.

Proteolysis post-mortem is governed by calcium-dependent proteases, μ- and m-calpain, and its inhibitor, calpastatin [31]. Calpain is responsible for the breakdown of several myofibrillar proteins, which results in beef tenderization [32,33]. However, if the muscle structure is deeply affected by the cold regime, resulting in excessive sarcomere shortened by the overlap of thin and thick filaments, proteolysis will not be able to efficiently disrupt the architecture, and the beef will not tenderize, even after a long period of aging [34]. The rate of temperature and pH declines depends on muscle anatomic location in the carcass and in the fat distribution covering those muscle masses. In this context, longer sarcomere length in the TB of excitable cattle (Figure 7) observed in the present study was not expected, although it could partially explain the observed differences in the MFI of these cattle. Aligned with the possible greater calcium release due to greater porphyrin metabolism in excitable cattle, for both muscles, MFI started at a faster rate (Figure 8A,B). For TB, for example, longer sarcomere and early calcium release from the sarcoplasmic reticulum were sufficient to result in the difference observed at 7 d of aging. Additionally, the faster pH decline in TB from excitable cattle could also positively contribute to early calpain-1 activation, once decreasing ATP concentrations and acidic conditions can lead to sarcoplasmic reticulum dysfunction, increasing sarcoplasmic Ca^2+^ [35]. Although the fragmentation was faster within the TB of excitable cattle, it reached a plateau sooner when compared to the pattern in the TB of calm animals. For LL, on the other hand, although no differences were observed at 1 and 7 d of aging, beef of excitable cattle reduced the rate of fragmentation, while the beef of calm animals fragmented more. In this case, after 28 d of aging, the MFI was greater (*p* = 0.076) in LL of calm compared to LL of excitable cattle, which would be associated with more tender beef. Increased stress response or excitable temperament was associated with tough beef, which could be the result of the persistence of the intact form of calpastatin [36]. Additionally, the greater accumulation of taurine early post-mortem, as well as the myo-inositol/glycine/glycerol 24 h in LL of excitable cattle, are evidence of potentially greater activation of a protein kinase A phosphorylation cascade, which increases calpastatin inhibitory activity [37].

## 5. Conclusions

The inherent slaughter stress response can differently affect cattle that, throughout their lives, modulate the sympathoadrenal system and hypothalamic-pituitary-adrenal axis, which results in cellular modifications regarding energy balance. Taurine at 1h post-mortem in LL of excitable cattle, along with glycine in both muscles at 24 h post-mortem, were evidences of this modified muscle metabolism in a differential stress response model. Additionally, the myofibrillar fragmentation differences observed for the LL muscle among the groups of temperament are not driven by the pH or temperature declines. In fact, the slower rate and lower extension of fragmentation in LL of excitable cattle contribute to the altered physiological condition of those animals. Finally, in excitable cattle, the stress response within skeletal muscle cells contributes to up-regulation of mitochondrial function, as well as promoting cellular conditions to post-translational modifications that will decrease myofibrillar fragmentation.

## Data Availability

The data presented in this study is available upon request to the corresponding author.
